# Jobs and Punishment: Public Opinion on Leniency for White-Collar Crime

**DOI:** 10.1177/10659129231176211

**Published:** 2023-05-19

**Authors:** Simon St-Georges, Vincent Arel-Bundock, André Blais, Marco Mendoza Aviña

**Affiliations:** 1 5622 Université de Montréal, Montreal, QC, Canada; 2 1812 Harvard University, Cambridge, MA, USA

**Keywords:** public opinion, criminal law enforcement, white-collar crime, corporate leniency, deferred prosecution agreements

## Abstract

Governments routinely offer deals to companies accused of white-collar crimes, allowing them to escape criminal charges in exchange for fines or penalties. This lets prosecutors avoid costly litigation and protects companies’ right to bid on lucrative public contracts, which can reduce the likelihood of bankruptcies or layoffs. Striking deals with white-collar criminals can be risky for governments because it could affect the perceived legitimacy of the legal system. This article explores the conditions under which the general public supports leniency agreements. Building on theoretical intuitions from the literature, we identify three characteristics that could affect mass attitudes: home bias, economic incentives, and retribution. We conduct a survey experiment in the United States and find moderate support for leniency agreements. Whether the crime occurs on US soil or abroad does not affect public opinion, and the number of jobs that would be jeopardized by criminal prosecution only has a small effect. Instead, survey respondents become much more supportive of a deal when it includes criminal charges for the corporate managers who were personally involved in the alleged wrongdoing. In the court of public opinion, punishing a handful of individuals appears to matter more than saving thousands of jobs.

White-collar crime is common, and its consequences are dire. As of 2021, financial losses from white-collar crimes are estimated at $426 billion to $1.7 trillion a year, with such a wide range of estimates due to most crimes being unreported (Flynn 2022). White-collar crime harms victims directly, but also affects the broader community: it can have environmental and security implications as well as hamper governments’ ability to fund public services (International Public Sector Fraud Forum 2020).

How the justice system deals with white-collar crime can affect its own legitimacy. Since the 2007–2008 financial crisis and the corporate crime scandals that followed, the public’s attitude toward law enforcement and the justice system has soured. In 2019, 41% of Americans held an unfavorable opinion of the Department of Justice. It ranked lower than other law enforcement bodies such as the FBI and the CIA, and lower than the U.S. Postal Service or the Environmental Protection Agency (Pew Research Center 2019). This low esteem may be linked to the widespread belief that criminals can often escape punishment if they are rich and well-connected (Claypool 2021).

In recent years, corporate scandals have remained salient, but the number of white-collar prosecutions and trials has continuously declined (TRAC 2021, 2022). Part of this decline can be traced to the rise of leniency deals such as “Deferred Prosecution Agreements.” These legal agreements are negotiated between public prosecutors and lawyers who defend companies accused of criminal wrongdoing. Prosecutors will typically end criminal court proceedings without a guilty plea if the accused company agrees to pay fines and implement good governance reforms. The intended goal of these agreements is to avoid costly legal proceedings and to allow companies to remain in business without the stigma and legal consequences that come with an admission of guilt. However, even when these deals impose large financial penalties, they often amount to one-tenth of one percent of a corporation’s market capitalization, and the stock price of public companies usually goes up after their announcement (Coffee Jr 2020; Markoff 2013). Leniency agreements, therefore, risk being perceived as too favorable towards corporations. Over-relying on them, in the absence of more orthodox criminal proceedings, might lead to the weakening of the punitiveness of law enforcement (Campbell 2019).

The widespread use of these leniency agreements, coupled with a decrease in corporate investigations and prosecutions, poses an important challenge to the legitimacy of corporate criminal law and the justice system. Indeed, legal scholars have been asking whether allowing white-collar offenders to escape criminal proceedings “undermine[s] the public’s confidence in the administration of justice” (Coffee Jr 2020, 36; Rakoff 2014). Indeed, according to many criminal law scholars, “there remains a deep public sentiment that corporations are ‘getting away’ with crime,” especially since the housing bubble burst (Buell 2016, 117).^1^ Yet, we have very little systematic evidence about public opinion on these matters, particularly when it comes to the factors shaping the acceptability of deals between the government and corporate actors.

This article addresses these concerns by taking a first step toward a better empirical understanding of the legitimacy and acceptability of different kinds of leniency agreements. To achieve this, we conduct a survey experiment in the United States and assess people’s views on different kinds of deals between public prosecutors and corporate actors. The results of this study yield key insights into the public acceptability and legitimacy of leniency deals.

We find that American citizens are moderately supportive of the government striking deals with white-collar criminals. Surprisingly, support for leniency agreements does not appear to be affected by the economic consequences of a guilty verdict (i.e., the number of jobs that would be lost), or by the location where criminal activity allegedly occurred (i.e., domestic or foreign). In contrast, respondents’ support for a deal increases considerably when criminal charges are pressed against the individual managers who were personally involved in criminal wrongdoing. This suggests that public opinion is driven more by a concern for punishment and retribution, rather than by a rational economic calculus to save jobs.

The rest of this paper unfolds as follows. First, we argue that the empirical legitimacy of the legal system is tied to public perceptions of both the severity and the policy response to white-collar crime. Second, we discuss prior research on public opinion on white-collar crime and suggest that there is a need for a better understanding of attitudes toward policy instruments like leniency agreements. Third, we consider theoretical arguments in favor and against leniency agreements and identify key characteristics which may affect the public’s view of this policy. Fourth, we describe a survey experiment designed to gauge the factors that affect the level of public support for striking deals with white-collar criminals. After reporting the results of this experiment, we conclude by discussing the implications of our findings for our understanding of public perceptions of white-collar crime and, more broadly, for the legitimacy of the legal system.

## Legitimacy, Public Policy, and Public Opinion

Western societies are going through “an age of distrust”: citizens increasingly exercise vigilance, oversight, and denunciation, which means power holders must either adapt to popular pressures or be subjected to the tribunal of public opinion (Rosanvallon and Goldhammer 2008). Like some other parts of the legal system, the Department of Justice has built a fair amount of autonomy over the years. Today, it holds a “reservoir of goodwill” that allows it to “get away with unpopular decisions (Gibson and Caldeira 2009, 140).” But this reservoir is not infinite. Even though public prosecutors and civil servants have a great amount of autonomy and executive discretion, they must be wary of their reputations in the public eye (Noyon, de Keijser, and Crijns 2020; see also Carpenter 2001, 2010).

As Tyler and Jackson (2013) note, the empirical legitimacy^2^ of the governmental organizations in charge of prosecuting white-collar crime is built on the trust that people bestow on the criminal justice system and the perceived obligation to obey it (see also Sunshine and Tyler 2003; Tyler 2009). This may be an especially sensitive area of action because negative news and unpopular decisions can erode public support and eventually compromise the legitimacy of both courts and law enforcement agencies (Casillas et al. 2011). As Zilis (2021) has argued in the case of the Supreme Court, widespread antipathy toward “big business,” and a string of unpopular decisions in favor of corporate rights, may have contributed to decreasing its empirical legitimacy. In the United States, the Department of Justice (DoJ) oversees the Fraud Section, responsible for investigating and prosecuting sophisticated economic crimes. As such, it seems useful to think about the handling of white-collar criminal proceedings, trials, plea deals, and leniency agreements as a key part of the DoJ’s mission, and efforts to foster its empirical legitimacy.

Political scientists and criminologists have shown that public officials care about public attitudes and preferences toward crime rates, high-profile cases, criminal sentencing, and policy responses to crime (Erikson et al. 2002; Pickett 2019; Shapiro 2011). In *Incarceration Nation*, for example, Enns (2016) shows how mass incarceration is in large part a political response to the public’s views on punitiveness, spending priorities, and perceptions of public safety. Legal officials at the state and federal levels are concerned with public opinion as well, especially since most of them are elected or appointed by elected officials (Pickett 2019, 418-419). More than half of US states have judicial elections for trial, appellate, and higher courts (Brace and Boyea 2008).^3^ Non-elected legal actors also tend to be responsive to public opinion because they are concerned about maintaining institutional legitimacy. Casillas et al. (2011) suggest that public opinion exerts an important influence on Supreme Court decisions. Owens and Wohlfarth (2017) find similar results for federal circuit courts, despite both levels of Courts granting lifetime tenure. Indeed, legal scholars recognize the need for sentences to be neither too harsh nor too lenient because public trust in legal institutions is central to the legitimacy of the law (Robinson 2013).

## Public Opinion on White-Collar Crime

Despite its major consequences, white-collar crime has often been portrayed as less harmful and worrying than violent street crime (Cullen et al. 2019). Indeed, early research suggested that white-collar crimes were mistakenly perceived as being of similar seriousness to “victimless and minor property offenses” (Rossi et al. 1974). This perception has evolved over the past few decades, with events like the savings and loan crisis, the Enron scandal, and the Great Recession.^4^ Some evidence suggests that the public may now view corporate crime as equally or more damaging than conventional street crimes (Michel 2016; Cullen et al. 2019). An overwhelming majority of Americans support stricter penalties, including longer prison terms, for executives who commit fraud (Unnever et al. 2008). Voters across party lines agree that the criminal justice system unfairly targets the poor over the wealthy, that corporations and wealthy people regularly evade fair punishment, and that this is a concerning issue for public trust and the rule of law (Smith 2021).

When it comes to how white-collar crime is prosecuted in the US, there seems to be a disconnect between the government and the people it represents (Grasso 2021). In the wake of corporate scandals, the public and politicians often demand severe sanctions for white-collar criminals (Healy and McGrath 2019).^5^ Yet, corporations or their managers are rarely accused of criminal conduct.^6^ In the few cases that are prosecuted, the DoJ increasingly resorts to leniency agreements (Garrett 2014, 2019). These agreements can conform to legal principles, but they may not always match the public’s idea of what white-collar criminal proceedings ought to look like.

White-collar crime enforcement is very limited, and most cases against offenders do not invoke criminal laws (Baer 2018, 90; Simpson et al. 2019). Only 3% of federal prosecutions target white-collar crime (Bajoka PLLC 2020). What’s more, since the terrorist attacks of 9/11, law enforcement resource allocation has shifted away from white-collar crime toward fighting terrorism (Nguyen 2021). White-collar enforcement declined to an all-time low during the Trump administration, which further shifted resources toward immigration surveillance (Garrett 2019; Hurtado et al. 2020; McGrath and Healy 2021). Current law enforcement practices may thus fail to have much of a deterrent effect on potential offenders and to sustain public trust in the criminal system (Claypool 2021, Smith 2021).

It is important to assess whether certain characteristics in crimes and ensuing legal proceedings shape views on punitiveness and fairness more than others (Van Slyke and Rebovich 2016). Among the public, white-collar crimes are perceived as less harshly prosecuted than other types of crimes; indeed, most people recognize that white-collar offenders have a smaller chance of being apprehended and punished compared to other types of criminals (Van Slyke and Rebovich 2016).

There is unfortunately little research pertaining specifically to public attitudes and white-collar crime enforcement (Healy and McGrath 2019). The research that does exist does not tell us how leniency for corporate offenders stands in public opinion compared to traditional sanctions or incarceration. There is thus a need to further examine public views about white-collar crime, particularly how the public perceives criminal sanctions for business entities and executives (Cullen et al. 2019, 224).

This article is part of an emerging research agenda that assesses public perceptions of white-collar crime and the policy responses to it, including the use of leniency agreements. The public considers white-collar crime to be serious, and both legal and political actors should be susceptible to respond to their views.

Given public mood on these matters, the acceptability of legal tools like leniency agreements ought to be considered, especially given their novelty and widespread use in white-collar crime cases. Studying leniency agreements allows researchers and policymakers to uniquely understand how the public perceives the trade-offs between punishing corporate wrongdoing and minimizing harm to innocent third parties, such as workers. This balancing of competing interests is at the heart of leniency agreements (Lund and Sarin 2022). As opposed to trials and plea agreements that both seek a corporate recognition of guilt, leniency agreements can be seen as a compromise of punishment, a way of prosecuting corporations without using the fullest extent of the law. Some members of the public may view leniency agreements as too lenient if they allow corporations to escape punishment only by paying fines. Others may view leniency as reasonable to avoid collateral damage when prosecuting a corporation, the traditional way would lead to important job losses. We discuss in more detail below how certain trade-offs and features of leniency agreements inform our hypotheses.

## When is leniency acceptable?

Leniency agreements are typically concluded in complex situations such as antitrust, bribery, or fraud cases under the Foreign Corrupt Practices Act, or to settle pharmaceutical, public health, or environmental accusations. Such cases can be extremely costly to prosecute. Striking a deal with white-collar criminals can thus be advantageous for resource-constrained governments, by allowing them to avoid costly legal proceedings. In so doing, leniency agreements can free-state resources for more enforcement actions, and the penalties paid by businesses under those agreements can also help capacity building. Furthermore, leniency agreements foster self-disclosure of corporate wrongdoings if they are only offered to businesses willing to collaborate with the authorities. These deals could ultimately increase the total number of enforcement actions against corporate crime (OECD 2019).

From the perspective of businesses, leniency agreements can also be beneficial, because they avoid the consequences of plea bargains and criminal trials, which involve an admission or a recognition of guilt (Garrett 2019, 15). The consequences can be severe: many countries have public contract laws that exclude companies with criminal convictions from getting public contracts.^7^ For many large companies, access to public contracts is necessary for competitiveness and survival. Lenders and suppliers may also cut contractual ties with businesses plagued by criminal scandals, which provokes cascading effects on the reputation and viability of the business.

Despite advantages for both business and government, deferred prosecution agreements remain a controversial policy instrument. Reilly (2018) writes that such deals make the criminal justice system a “mockery” because they allow discretion abuses while exacerbating inequalities between litigants. Leniency agreements are criticized on all sides of the political spectrum. Conservatives worry that they interfere with the rigorous application of the principles of law and order, while progressives fear that they may amount to a small corruption tax for large companies that are “too big to jail” (Garrett 2014; Steinzor 2015). Many critics do not oppose the existence of these deals altogether. Instead, they deplore their abuse by the Department of Justice, which they think should pursue more plea bargains and trials for offending businesses (Buell 2016; Eisinger 2018; Garrett 2014; King and Lord 2018; Koehler 2015). Some fear that leniency agreements may compromise the independence of prosecutors if they need to please the political officers of the Department of Justice or if they become too risk-averse to go to trial (Eisinger 2018; Uhlmann 2013). This can reduce their expertise and willingness to fight high-profile cases in courts, depriving both the judicial community and citizens of jurisprudential development and jury feedback (Campbell 2019, 283; King and Lord 2018, 117, 127). Risk-aversion and groupthink from prosecutors are one of the criteria that may delegitimate an agency such as the Department of Justice in the eyes of the public (Levi, 2013, 167).

In the empirical portion of this study, we draw inspiration from the main arguments in favor and against leniency agreements, to identify characteristics that could affect whether the general finds such agreements acceptable. We focus on the primary policy objectives of corporate accountability, including for individual wrongdoers, while also protecting jobs and business reputation. We also explore how a sense of nationalism may affect the acceptability of leniency agreements.^8^

### Individual Responsibility

According to many observers, deferred prosecution agreements are not deterrent enough because they permit too many senior executives involved in criminal accusations to escape prosecution (Coffee Jr 2020, 9, 32; Eisinger 2018; Garrett 2011, 2014). Garrett (2014, 13) confirms that only about one-third of the cases in which public companies benefited from deferred prosecution agreements led to criminal prosecutions of individuals. Furthermore, few higher-up officers were charged; most individual prosecutions targeted “middle” and “low” company employees (Garrett 2015, 1802).

When guilty players directly responsible for wrongdoing go unpunished, people might come to believe that the legal system is not working properly, and legitimacy might suffer. Facing mounting criticism, the Obama administration attempted to correct this situation before the 2016 general elections with the “Yates Memorandum,” named after the Deputy Attorney General at the time (Yates 2015). Essentially, the Justice Department modified its official policy on deferred prosecution agreements to stress the need for individual accountability. Despite these efforts, the number of individual prosecutions continued to decline. For example, of the 21 deferred prosecution agreements signed in 2018, only five involved individual prosecutions (Garrett 2019, see also Coffee Jr 2020, 37, 50). Given the rising controversy surrounding the lack of prosecutions of white-collar criminals, we expect citizens to be sensitive to this consideration. We expect that survey respondents will be more supportive of a leniency agreement if it guarantees that corporate managers personally involved in the wrongdoing will be prosecuted.

• Hypothesis 1: The public is more likely to support leniency deals when they specify that managers personally involved in the wrongdoing will face criminal charges.

### Economic Consequences

One of the strongest and most often cited arguments in favor of deferred prosecution agreements is that they protect third parties from the actions of individuals directly responsible for corporate crimes (Golumbic and Lichy 2014). By reducing the economic impact of a recognition of guilt on the corporation, they protect innocent employees, pensioners, or contractual partners from a criminal recognition of guilt (Ainslie 2006; Resnik and Dougall 2006; see, however, Markoff 2013). While criminal wrongdoing should warrant consequences for a company’s managers, targeting the whole company puts its employees in a precarious position for no fault of their own. Ensuring proper criminal law enforcement is key to the perceived legitimacy of the DoJ, but saving jobs is also a legitimate economic and political goal for governments.

Our research design incorporates these concerns for the protection of American jobs. Specifically, it ascertains whether the level of support for a deal varies depending on the number of employees of the accused corporation. We expect acceptability to increase when a higher number of jobs are at risk. Americans are concerned with policies that generate negative economic consequences and are willing to go to great lengths to preserve even a handful of jobs (Mutz and Lee 2020). For example, policy preferences over globalization and climate change are highly sensitive to local job market outcomes (Feng et al. 2021; Mansfield and Mutz 2013; Scheve and Slaughter 2001; Vona 2019).

Considering how important job creation is for electoral outcomes (Lewis-Beck and Stegmaier, 2000) and how sensitive citizens are to economic shocks affecting employment (Margalit 2019), we expect that survey respondents will be more supportive of leniency agreements if they protect the livelihoods of innocent workers.

• Hypothesis 2: The public is more likely to support leniency deals when they protect a higher number of jobs.

### Home Bias

We chose bribery and fraud for the context of our research design because such scenarios allow us to consider the international dimension of crime. The rise of deferred prosecution agreements in America has had an interesting side-effect: prosecutors are harsher on non-US corporations than on US corporations involved in criminal activities. The Department of Justice indicts more often foreign-based companies compared to domestic companies; when given the option to enter a leniency agreement, foreign companies are made to pay considerably higher fines (Garrett 2011, 2014). By contrast, prominent Department of Justice cases involving US-based public companies and financial institutions are less common (Garrett 2019). This phenomenon contributed to the adoption of leniency agreements in other countries, partly because other law enforcement agencies collaborated with, or reacted to the international reach of the Department of Justice. Leniency agreements spread throughout the Western world and even Asia, notwithstanding significant differences in the countries’ criminal justice systems (OECD 2014, 2019). National economic interests and patriotism may explain this phenomenon (Saint-Martin 2021; see also Lüth 2021; Woll 2023). It is important to understand if a national or patriotic sentiment also exists in the population concerning punitive attitudes. We examine whether US citizens exhibit a home bias as follows. We compare, on one hand, their level of support for leniency deals when the crime is committed domestically against the US government and, on the other hand, when the crime is committed abroad against a foreign country and its foreign citizens.^9^ We expect the public to be less lenient with a corporation that defrauds the US government instead of a foreign government. Therefore, we expect citizens to be more supportive of a leniency agreement when fraud and corruption are committed abroad.

Previous studies suggest the presence of a home bias: people favor their own country and their fellow citizens. This is a consistent finding in research bridging public opinion and international political economy. On trade, Mutz and Lee (2020) find that American citizens systematically value their co-nationals’ livelihoods more than those of other citizens living in partner countries; similarly, Brutger and Rathbun (2021, 880) find that “Americans have an egoistically biased sense of fairness, responding particularly negatively to any outcome that leaves the United States relatively worse off.” On outsourcing, Mansfield and Mutz (2013) argue that Americans see economic exchanges as a zero-sum game between their domestic ingroup and foreign outgroups. On international corporate taxation, Arel-Bundock and Blais (2023) find that the public favors reforms to their own country’s financial advantage. On foreign aid, Milner and Tingley (2013) find that many Americans only support foreign aid when it strategically aims to benefit the US economy. We expect that people will be less lenient when fraud is committed against the US government than when the fraud affects a foreign government.

• Hypothesis 3: The public is more likely to support leniency deals when the crime is committed against a foreign government rather than their own government.

To study these issues, it is useful to focus on a particularly salient category of leniency agreements that have caused controversy in recent years. Deferred prosecution agreements follow legal frameworks that allow for variation along the dimensions described above. In the next section, we describe a survey-based research design which allows us to test our three hypotheses in the context of deferred prosecution agreements. Centering our research design on these agreements increases external validity by allowing us to link our findings to concrete cases of prosecutorial leniency in white-collar cases.

## Research Design

The theoretical and substantive considerations explored above led us to study the effect of three factors on public support for deferred prosecution agreements: (1) whether the deal includes criminal charges for individuals involved in the crime; (2) the number of jobs jeopardized by the prosecution; (3) whether the US or a foreign government was targeted by the criminal wrongdoing. To measure how these three factors relate to acceptability, we conducted a pre-registered survey experiment with an online sample of 2000 US adults.^10^

We set our study in the context of corruption and fraud against a government, which typically implies an illegal exchange of favors with a high level of violation of public trust and a large pool of victims (Cullen et al. 2019). When bribery, fraud, or corruption occurs, the local population pays much higher costs for public projects, often with increased environmental and security risks (International Public Sector Fraud Forum 2020). Moreover, when a corruption scandal irrupts, the loss of trust in institutions can lead to further economic and social consequences.

The experiment consists of a single vignette about a fictional company:

*We would like to have your views about deals that are sometimes made between the U.S. Department of Justice and American companies accused of criminal activity.*

*Imagine that an American company is involved in a major case of bribery and fraud against the [U.S. government / government of Brazil]. The U.S. Department of Justice has strong evidence that the company committed this crime. If they go to court and the company is found guilty, the company will be forced to declare bankruptcy and lay off [100 / 5,000] innocent American employees.*

*To save those jobs, the U.S. Department of Justice decides to offer the company a deal: The lawsuit will be dropped if the company agrees to pay a big fine. [In addition, 4 of the company’s managers will face criminal charges for their personal involvement in the bribery.]*

To assess how each of the three factors above relates to acceptability, we introduce random variation among three factors in the vignettes that respondents read. Each of those factors is randomized independently.

For the *Criminal Charges* factor, half of the respondents are assigned to read that “4 of the company’s managers will face criminal charges for their personal involvement in the bribery” at the end of the vignette. The other half does not see this sentence.

For the *Jobs at Risk* factor, half of the respondents are assigned to read that 100 jobs are at risk, whereas the other half are told that 5000 jobs are at risk.

For the *Crime Abroad* factor, half of the respondents are assigned to read that the American company committed the crime on US soil, whereas the other half read that an American company committed the crime in Brazil.

After displaying the vignettes, we measure the outcome of interest with the following question:

*Do you support or oppose this deal? 0 means that you “Strongly Oppose” the deal. 10 means that you “Strongly Support” it.*

Now that the research design has been outlined, the following section examines the data and presents the results.

## Results

Before describing how the randomized treatments relate to support, it is helpful to take an exploratory look at the outcome variable. Table 1 shows the average level of support for a deal and its standard deviation for different subgroups of the survey sample. Several points are noteworthy.

**Table 1.** Support for Deferred Prosecution Agreements on A Ten-Point Scale by Gender, Age Group, and Education Level.

	Mean	Std. Dev.
Gender		
Man	6.44	2.66
Other	4.67	1.97
Woman	5.98	2.53
Age		
18–34	6.45	2.49
35–54	6.27	2.64
55+	5.67	2.58
Education		
No formal education	5.90	1.66
Some primary school	6.43	2.92
Primary school completed	6.52	2.51
Some secondary/high school	6.27	2.45
Secondary/high school completed	6.03	2.45
Some college or university	6.25	2.59
University completed or higher	6.30	2.79

To begin, we see that the average level of support for a deal is somewhat positive among the American public, hovering above 6 on a scale ranging from 0 to 10. However, we also see that there is a fair amount of variation across that mean, with a standard deviation of about 2.5. Table 1 also shows substantial variation in support across socio-demographic groups. On average, men are more supportive of a deal than women, and the level of support appears to decline with age.^11^ In contrast, there does not appear to be any clear relationship between education and this item.

To estimate the association between experimental conditions and support, we estimate a linear regression model of this form^12^

$$\text{Support}\;\text{Deal}=\beta_{0}+\beta_{1}\text{Criminal}\;\text{Charges}+\beta_{2}\text{Jobs}\;\text{at}\;\text{Risk}+\beta_{3}\text{Crime}\;\text{Abroad}+\varepsilon$$

$\beta_{1}$ estimates the effect of the *Criminal Charges* treatment, which is equal to 1 when respondents learn that the company’s managers will face personal criminal consequences for their actions, and 0 otherwise. If people are motivated by a desire for punitiveness, they should be more willing to support a deal that punishes white-collar criminals, and the $\beta_{1}$ coefficient should be positive. $\beta_{2}$ estimates the effect of the *Jobs at Risk* treatment, which is equal to 1 if 5000 American jobs are at risk, and 0 if 100 jobs are at risk. Since respondents should be more lenient when the costs of prosecution increase, we expect the $\beta_{2}$ coefficient to be positive. $\beta_{3}$ estimates the effect of the *Crime Abroad* treatment, which is equal to 1 if the crime was committed in Brazil and 0 if it occurred in the US. We expect American respondents to be more favorable to a deal when the company commits a crime abroad rather than on US soil. If this is correct, the $\beta_{3}$ coefficient should be positive.

**Figure 1.**
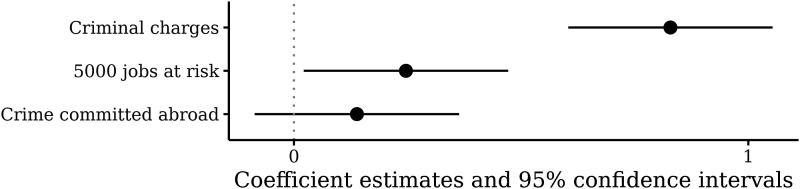
The effect of criminal charges, jobs at risk, and crime location on support for deferred prosecution agreements. The dependent variable is on a scale of 0–10, where 10 means “strongly support” the deferred prosecution agreement. The outcome has a mean of 6.2 and a standard deviation of 2.6.

Figure 1 presents our point estimates along with 95% confidence intervals built using heteroskedasticity-consistent standard errors. The full results are presented in Table B of the online appendix.

## Discussion

The first major result of our study is that the inclusion of criminal charges has a large effect on public support for such agreements. On average, pressing criminal charges against four of the company’s managers increases support for a deal by about 0.8 on the ten-point scale, or about 1/3^rd^ of a standard deviation ( p<0.001 ). This is a substantial effect and a key finding of this study.

The second important result in Figure 1 is that the number of jobs has a positive but very small effect on acceptability. On average, moving from 100 to 5000 jobs at risk increases the level of support for a deal by 0.25 on a ten-point scale. While this result crosses conventional thresholds of statistical significance, the estimated effect size is substantively small: It is equivalent to about 1/10^th^ of a standard deviation in the dependent variable. This weak effect is all the more striking given that the treatment is arguably very strong; the difference between 100 and 5000 lost jobs should be an important consideration for most citizens and policymakers (see for ex. Feng et al. 2021; Mutz and Lee 2020).

The third important result is that whether the US or a foreign government is targeted does not appear to be an important consideration for respondents. Indeed, $\beta_{3}$ is substantively small and it is not statistically significant. There is no statistically significant effect of the location of a corporate crime on people’s propensity to support a deal. This is also surprising given previous research on national economic interest and patriotism in public attitudes (see e.g., Brutger and Rathbun 2021; Mansfield and Mutz 2013).

These findings are striking; they point to a major tension between economic rationality and hunger for punishment. For many American respondents, it is more important to punish four (4) guilty managers than save 5000 innocent workers. Our results also suggest that American patriotism does not affect the acceptability of leniency agreements. These results send a strong signal to practitioners and politicians that the legitimacy of white-collar crime enforcement rests more on appropriate punishment than on pragmatic, economic considerations.

Democratic theory and consensus perspectives on criminal law suggest that crime control must reflect public wishes and important social norms (Van Slyke and Rebovich et al. 2016). The criminal justice system plays a key role in American society, but its handling of corporate criminal cases in the 21^st^ century may contribute to weakening its legitimacy. To preserve its legitimacy, public administrations must respond to public opinion on corporate crime levels and sentencing (Tyler and Jackson 2013; see also Moore and Mills 1990; Roberts and Stalans 2004; Unnever et al. 2008, 165). At a time when politicians and the public frequently condemn corporate excesses and criminal conduct (Healy and McGrath 2019), leniency agreements for white-collar criminals risk diminishing the public’s trust in its institutions.

Our study sheds new light on these matters. Using a vignette experiment, we measure how variations in key features of a deal affect support by the public. Our results suggest that even though public acceptance of leniency agreements is moderately positive, respondents do not give much weight to the economic rationale—protecting jobs—that often underlies them. Instead, acceptance is considerably enhanced when prosecutors prioritize holding company managers accountable for corporate crimes. Moreover, respondents are indifferent to the location of the crime: their opinion does not change when a foreign country rather than the US is targeted by bribery and fraud.

Taken together, our results suggest that people support leniency agreements when they perceive them as fair and sufficiently punitive—regardless of how many US jobs they can save. Our results thus suggest that deontological considerations trump utilitarian cost-benefit calculations in the perceived legitimacy of these deals. Research in moral psychology indicates that “political attitudes held with moral conviction are associated with a characteristically deontological (action-oriented) processing style,” which implies disregard for the practice of weighing costs against benefits (Ryan 2019, 429). Deontology, in this sense, refers to “the psychological mode in which judgments stem from the inherent appropriateness of an action, rather than consequences” (Ryan 2019, 428). Therefore, respondents who think about an issue using a deontological processing style will tend to be insensitive to information about its policy effects.

A clear takeaway from this study, for practitioners and researchers alike, is that the public seems comfortable with leniency agreements when they ensure criminal prosecutions for influential individuals. These results are in line with the official statement from a former chief prosecutor of foreign bribery, Mark Mendelsohn, according to whom “It is [the Department of Justice’s] view that to have a credible deterrent effect, people have to go to jail” (Corporate Crime Reporter 2008). If public officials care about public opinion as much as they claim they do, leniency agreements aimed at protecting innocent parties should also prioritize the inclusion of criminal charges allowing for the punishment of guilty ones.

It is of utmost importance for the legitimacy of the legal system that white-collar criminals do not go unpunished. Accusations against corporate entities rarely lead to consequences for high-profile executives. There was no individual criminal accountability for officers of Boeing, concerning fraud conspiracy over the 737 MAX evaluations, Wells Fargo, regarding its predatory cross-selling fraud, or even Purdue Pharma for its role in an illegal opioid scheme (Claypool 2021). These cases are not exceptional since individual prosecutions accompany only 27% of leniency agreements (Garrett 2019). Yet there is evidence suggesting that optimal corporate deterrence should focus more on seeking consequences for top individuals and less on financial penalties for companies (Lund and Sarin 2022).

Because the perceived legitimacy of the Department of Justice, and other law enforcement agencies, largely depend on public perceptions of fairness, more research is required on corporate crime enforcement in general. Public attitudes toward corporate crime enforcement may notably vary depending on how and when respondents are probed. They may react differently to similar experiments in periods of economic crisis, or in the face of corporate scandals involving large institutions, such as Enron, WorldCom, Halliburton, and major banks during the Great Recession. Survey and experiment results may also be influenced by the level of awareness of criminal corporate enforcement, and how its rather technical concepts are presented to respondents (see Michel et al. 2016a, 2016b). More political science research should also focus on public support for law enforcement. Our results differ from expectations on the impact that patriotism and national economic interests may have in shaping public attitudes (Arel-Bundock and Blais 2023; Brutger and Rathbun 2021; Feng et al. 2021; Mansfield and Mutz 2013; Milner and Tingley 2013; Mutz and Lee 2020; Scheve and Slaughter 2001; Vona 2019).

## Supplemental Material

Supplemental Material - Jobs and Punishment: Public Opinion on Leniency for White-Collar Crime Click here for additional data file. Supplemental Material for Jobs and Punishment: Public Opinion on Leniency for White-Collar Crime by Simon St-Georges, Vincent Arel-Bundock, André Blais, and Marco Mendoza Aviña in Political Research Quarterly

## Notes

1. Such criticisms have reached the general public via popular culture, through TV shows and documentaries like “Dirty Money” (Teodorczuk 2018; see also Khardori 2022; Viswanatha and Michaels 2021).

2. The concept of “empirical legitimacy” refers to the perceived right to rule (de Fine Licht et al. 2022). It can be contrasted with “normative legitimacy,” which focuses on the moral grounds to rule, such as justice or rationality (see Beetham 2013; Hinsch 2010; Merquior 1980).

3. Chief prosecutors, state, county, and district attorneys are, for the most part, elected or appointed by elected members of the executive branch (Wright 2008). Elected officials are more likely to respond to public mood on law and order, including oversight priorities (Erikson et al. 2002; Enns 2016).

4. Research in the 80s notably found that 89% of respondents believed white-collar criminals deserve to go to jail for their crimes like everyone else (Cullen et al. 1983). In 2005, one-third of respondents to a national survey regarded white-collar crimes as “more harmful to society than ordinary crimes,” while the majority of respondents thought “the government should allocate equal or more resources to tackling white-collar crime” (Holtfreter et al. 2008).

5. The term “white-collar crime” was coined in 1939 to distinguish it from “common” or “street” crime, which is often violent (Sutherland 1983). Even if definitions of white-collar crime are plural, they usually refer to “illegal activities that principally use deceit, deception, concealment, fraud, or misrepresentation to obtain money, property, or some other advantage, or to conceal or cover up other wrongdoing” (Strader et al. 2021). Therefore, white-collar crime encompasses a variety of crimes ruled by federal and state statutes on securities fraud, antitrust violations, bribery, tax offenses, bank embezzlement, postal and wire fraud, false claims and statements, and credit or lending fraud (Healy and McGrath 2019). White-collar crime is performed by legal entities, employees, but also rogue individuals. On the other hand, “corporate crime” refers to a corporation’s punishable conduct or of its representatives acting on its behalf (Braithwaite 1985).

6. For example, when reviewing the Standard and Poor 3000 between 1996 and 2013, Simpson et al. (2019) found that of the 10,812 cases brought against firms for accounting, bribery, environmental, and anti-competitive offenses, less than 5% opted for the criminal law venue. Public prosecutors instead favored administrative sanctions (57%) and civil suits (38%), despite having criminal prosecution as a clear statutory option for these kinds of cases. On average, criminal accusations target smaller, younger, and less influential companies or outright criminal organizations (Simpson and Yeager 2015; Simpson 2019).

7. For example, the US, China, Germany, Australia, Brazil, Canada, South Africa, and the World Bank all have such policies in place (World Bank 2021).

8. More features of deferred prosecution agreements could be studied in future research, such as the gravity of the crime committed, voluntary disclosure by the accused, awards to repeat offenders, the extent of judicial revision, or the importance of reparation to victims. According to Dodge (2020), white-collar crime traditionally leads to little compensation or justice for the victims. Leniency agreements also very rarely compensate victims of bribery and corruption in foreign countries (Garrett 2014; OECD 2019, 97).

9. Because our research design also allows us to examine reactions to the number of US jobs at risk, we do not consider the home bias associated with the foreign/domestic nationality of the accused corporation.

10. The pre-analysis plan was submitted to the AsPredicted website on 05/27/2021. Data were collected via the Qualtrics platform between May 31 and June 3, 2021. Participants were recruited by research firm Dynata to fill demographic quotas for gender (men and women), age group (18–34, 35–49, 50–64, 65+) education level (no high school, high school/some college, bachelors/postgraduate degree), and Census region (Midwest, Northeast, South, West). Since respondents who did not meet these quotas were systematically redirected out of our survey, our sample is balanced across these demographics without the need for survey weights. Table A of the online appendix compares our sample to data from the 2021 Current Population Survey by the US Census Bureau.

11. The relationships between support for the deal and gender, and support for the deal and age, are statistically significant at the α=0.001 level.

12. In Table C of the online appendix, we show that our results are not substantially different when we estimate an ordered logistic regression model instead of linear regression.
