# Supplementary material for: Jobs and Punishment: Public Opinion on Leniency for White-Collar Crime
Source: Polit Res Q. 2023 May 19;76(4):1751–63. doi: 10.1177/10659129231176211 (PMC10615619; doi:10.1177/10659129231176211)
Supplement: Supplemental Material - Jobs and Punishment: Public Opinion on Leniency for White-Collar Crime [file sj-pdf-1-prq-10.1177_10659129231176211.pdf]

Online Appendix

Table A: Comparing the sample to the U.S. Census 2021 Current Population Survey Based on the Sociodemographic Quotas Used for Participant Recruitment

Variable	Group	CPS	Sample
Age	18-27	29.25	30.18
	28-37	24.23	24.61
	38-47	24.49	24.21
	58-100	22.03	21.00
Gender	Man	48.49	48.52
	Woman	51.51	51.48
Education	No high school	9.60	12.43
	High school/Some college	55.38	54.54
	4-year degree/Postgrad	35.02	33.03
Region	Midwest	20.63	21.10
	Northeast	17.19	18.25
	South	38.27	38.60
	West	23.91	22.06

Table B: The dependent variable is on a scale of 0 to 10, where 10 means "Strongly support" the Deferred Prosecution Agreement. The outcome has a mean of 6.2 and a standard deviation of 2.6.

	Model 1
(Intercept)	5.59***
	(0.12)
Criminal charges	0.83***
	(0.11)
5000 jobs at risk	0.25*
	(0.11)
Crime committed abroad	0.14
	(0.11)
Num.Obs.	2001
R2	0.028
R2 Adj.	0.027
AIC	9452.5
BIC	9480.5
Log.Lik.	-4721.245
F	18.844
Std.Errors	HC3

* p < 0.05, ** p < 0.01, *** p < 0.001

Table C: Ordered logit model with intercept terms omitted. The dependent variable is on a scale of 0 to 10, where 10 means ``Strongly support" the Deferred Prosecution Agreement. The outcome has a mean of 6.2 and a standard deviation of 2.6.

	Model 1
Criminal charges	0.566***
	(0.079)
5000 jobs at risk	0.173*
	(0.078)
Crime committed abroad	0.086
	(0.078)
Num.Obs.	2001
AIC	8953.6
BIC	9026.4
RMSE	6.64

+ $p < 0.1$, * $p < 0.05$, ** $p < 0.01$, *** $p < 0.001$
